# Taxonomic Validation and Southern Range Expansion of *Campsomeriella whitelyi* (Kirby, 1889) (Hymenoptera: Scoliidae: Campsomerini) in Agricultural Landscapes of North-Central Chile

**DOI:** 10.3390/insects17070674

**Published:** 2026-06-28

**Authors:** Macarena González-Dossi, Fermín M. Alfaro, Elizabeth V. Villalobos, Jaime Pizarro-Araya

**Affiliations:** 1 Carrera de Ingeniería Agronómica, Escuela de Agronomía, Universidad de La Serena, Casilla 554, La Serena, Chile; macagdossi@gmail.com; 2 Laboratorio de Entomología Ecológica (LEULS), Departamento de Biología, Facultad de Ciencias, Universidad de La Serena, Casilla 554, La Serena, Chile; fmalfaro@userena.cl (F.M.A.); elivvillalobos@gmail.com (E.V.V.); 3 Programa de Doctorado en Biología y Ecología Aplicada, Universidad de La Serena, Casilla 554, La Serena, Chile; 4 Programa de Doctorado en Conservación y Gestión de la Biodiversidad, Facultad de Ciencias, Universidad Santo Tomás, Santiago 8370003, Chile; 5 Instituto de Ecología y Biodiversidad (IEB), Santiago, Chile

**Keywords:** parasitoid wasps, semi-arid agroecosystems, biological control potential, ecological niche modeling, agroecological services

## Abstract

The family Scoliidae is composed of parasitoid wasps of notable ecological and agronomic importance, particularly for their role in the natural control of soil-dwelling beetle larvae within agroecosystems. This study provides the first record of *Campsomeriella whitelyi* (Kirby, 1889) in Chile, a species originally described from the Tambo Valley, Arequipa, Peru. The specimens analyzed, previously identified as *Campsomeris servillei* (Guérin-Méneville, 1831), were found to correspond to *Campsomeriella whitelyi*, whose known distribution in Chile was restricted to the extreme north. Their identity was confirmed through morphological analysis, which revealed the presence of a distinct yellow band on the fourth abdominal tergite and an elongated posterior tibial spur—diagnostic characters consistent with the original description of the species. This record from the Coquimbo Region represents the southernmost known expansion of the species. Specimens were collected between 2017 and 2025 in horticultural and rainfed agroecosystems associated with the Elqui River Basin (Coquimbo Region, Chile), using entomological nets in targeted sampling efforts. The edaphoclimatic conditions of the area—characterized by light-textured soils, winter humidity, and a high availability of hosts—appear to have favored the establishment of this wasp in a previously unreported environment. Through MaxEnt modeling, areas of high environmental suitability were identified in Chile’s Norte Chico region. From an agronomic perspective, this finding opens opportunities to incorporate *Campsomeriella whitelyi* as a functional component in integrated pest management (IPM) programs, particularly in the biological control of Scarabaeidae (Coleoptera) larvae that affect root, bulb, and minor fruit crops. Its adaptation to semi-arid agricultural environments suggests potential resilience under climate change scenarios, as well as a low impact on non-target species. This study contributes to applied entomology and functional conservation, promoting the integration of beneficial Hymenoptera into sustainable agricultural systems of north-central Chile.

## Simple Summary

Correct species identification is essential for understanding insect biodiversity and for the effective management of agricultural ecosystems. Scoliid wasps (Hymenoptera: Scoliidae) are parasitoids of soil-dwelling beetle larvae and play an important role as natural enemies of agricultural pests. In this study, we report the first confirmed records of *Campsomeriella whitelyi* in north-central Chile, correcting previous misidentifications and significantly extending the known southern distribution of the species. Using detailed morphological analyses and environmental suitability modeling, we show that this wasp is able to establish in semi-arid agricultural landscapes characterized by irrigated crops and sandy soils. Our results highlight the importance of taxonomic validation combined with ecological modeling to better understand species distributions and their potential functional role in agroecosystems. This information contributes to improving biodiversity knowledge and supports the incorporation of native parasitoid wasps into sustainable agricultural management strategies.

## 1. Introduction

The order Hymenoptera is among the most diverse and functionally significant groups of insects in terrestrial ecosystems. Their contribution spans a broad range of ecosystem services, including pollination, nutrient recycling, the biological regulation of pest populations, and the improvement of soil structure [1,2]. These functions position hymenopterans as key agents of stability and resilience within both natural and agricultural ecosystems. In Chile, Hymenoptera constitutes the third most species-rich order of the class Insecta, with high diversity recorded in temperate and semi-arid zones [3]. Nevertheless, the richness remains insufficiently documented, particularly in ecologically transitional environments, such as the agricultural landscapes of the Norte Chico region [4,5].

Within the order, the family Scoliidae comprises solitary parasitoid wasps, commonly known as “scoliid wasps”, which play a prominent role as natural enemies of edaphic scarab beetle larvae (Coleoptera), which are subterranean pests of major agricultural importance. Females actively search for soil-dwelling hosts, which they paralyze and use for oviposition, allowing larval development as lethal ectoparasitoids [6,7]. Males, in contrast, are more commonly associated with mate-searching activity and floral resource use. Adult scoliid wasps frequently visit flowers, contributing secondarily to the pollination of both wild and cultivated plant species [8]. Consequently, scoliid wasps represent a functionally important group that may provide complementary ecosystem services through biological control and pollination support [9].

In particular, the tribe Campsomerini (subfamily Scoliinae) has been the focus of numerous systematic reviews worldwide, leading to the recognition of new genera and species across various regions [10,11]. In Chile, however, knowledge of this group has historically been limited, with records restricted to a single species from the extreme north of the country. This disjunct distribution pattern has been attributed to factors such as low ecological connectivity, restrictive climatic conditions, and limited entomological sampling efforts in intermediate zones [12].

At the same time, several cases of deliberate or accidental introductions of scoliid wasps for biological control purposes have been reported worldwide, particularly against larvae of *Phyllophaga* beetles [13]. Exotic species of *Campsomeris*, for instance, have been released in North America and other tropical regions, yielding mixed results in terms of efficacy, host specificity, and ecological safety [7]. These experiences have raised concerns due to the limited knowledge of host specificity, the potential impacts on native beetle and hymenopteran communities, and the ability of introduced species to establish beyond target areas. In this context, there is a growing need to study, conserve, and promote native species that provide key ecosystem services in agroecosystems. This is particularly important in semi-arid regions vulnerable to climate change [14].

In this scenario, the discovery of scoliid wasps in coastal agroecosystems of Chile’s Norte Chico raises important taxonomic, biogeographic, and functional questions, particularly regarding their specific identity, origin, ecological affinities, and potential role as providers of ecosystem services. Their presence in intensively managed systems under semi-arid conditions may be associated with both natural range expansion and passive anthropogenic dispersal mechanisms, such as movement of soil or agricultural materials carrying contaminated hosts [15].

This study seeks to validate the taxonomic identity of the scoliid specimens collected in the Coquimbo Region and characterize their recent distribution in Chile’s Norte Chico, considering relevant ecological and bioclimatic variables. It further discusses their potential functional role within semi-arid agroecosystems and their integration into applied conservation strategies and the sustainable management practices for entomological biodiversity.

## 2. Materials and Methods

### 2.1. Study Area

The study was conducted in the lower reaches of the Elqui Valley Basin (Coquimbo Region, Chile’s Norte Chico). This area is characterized by the intensive use of land for horticultural crops under modern irrigation systems. The climate is warm temperate with a prolonged dry season, classified as coastal semi-arid according to the Köppen–Geiger system, with an average annual temperature of 14.7 °C and rainfall concentrated between May and August, reaching annual totals of 80–150 mm [16].

From an edaphic perspective, the soils are well-drained Entisols with sandy-loam texture, conditions that favor the activity of rhizophagous scarab beetles, the principal host of scoliid wasps. Plant communities next to agricultural lands correspond to a mosaic of coastal sclerophyllous scrub and ruderal vegetation without significant human disturbance.

Sampling sites were distributed across the study area, with distances between sites ranging from a few meters to approximately 4 km (Table 1). Specimen collections were conducted in active fields cultivated with beet (*Beta vulgaris*, Linnaeus, 1753), potato (*Solanum tuberosum* Linnaeus, 1753), fava bean (*Vicia faba* Linnaeus, 1753), and Swiss chard (*Beta vulgaris* var. *cicla* Linnaeus, 1753), providing a realistic productive context for the occurrence of natural enemies.

### 2.2. Sampling and Taxonomic Analysis

Sampling campaigns were conducted seasonally between June 2017 and April 2025, with increased effort during the spring and summer months, when adult scoliid activity is typically highest. At each sampling event, active collections were performed along standardized 50 m transects for approximately 30 min per transect using entomological nets. Multiple transects were surveyed per site depending on field size and habitat heterogeneity, including crop margins, internal paths, and adjacent vegetation patches. Sampling intensity was maintained consistently among sites to ensure comparability of records. Captured specimens were preserved in 70% ethanol and subsequently mounted on entomological pins, following standard preservation protocols for Hymenoptera. Taxonomic identification was based on external morphological characters examined under a Leica EZ3 HD stereomicroscope (Leica Microsystems, Wetzlar, Germany), with reference to specialized keys for South American Scoliidae [6,10,17,18,19]. A total of 15 specimens were examined, including 7 males and 8 females. Species identification was based on morphological characters observed in both sexes. Diagnostic characters such as the shape of the posterior tibial spur, the morphology of the submarginal vein, and body pilosity were analyzed. Species identity was confirmed by direct comparison with type material housed in Chile’s National Natural History Museum (MNNC). All analyzed specimens were deposited in the scientific collection of the Laboratory of Ecological Entomology, Universidad de La Serena (LEULS) [20].

### 2.3. Potential Distribution Modeling

To assess the environmental suitability of *Campsomeriella whitelyi* in Chile’s Norte Chico and project potential areas of establishment, an ecological niche model was developed using MaxEnt v3.4.3 software, which operates under the maximum entropy principle [21]. The model produces a continuous map of environmental suitability, with cell values ranging from 0 (lowest suitability) to 1 (highest suitability). Multicollinearity among environmental predictor variables was assessed using the variance inflation factor (VIF). An iterative variable selection procedure was applied, whereby predictors with VIF values exceeding a predefined threshold (VIF > 5) were sequentially excluded. A random partition of the occurrence data was used, allocating 75% of the records for model training and 25% for testing. Fifty bootstrap replicates were performed, and overfitting was controlled using *β* = 1 [22]. A set of 19 bioclimatic variables from the WorldClim v2.1 database (Table 2), with a spatial resolution of 30 arcseconds (~1 km^2^), were used as independent predictors [23].

Layer preprocessing and spatial data management were performed in the R environment (v5.4.1) [24], using the raster package. Model outputs were visualized and georeferenced in QGIS v3.43, classifying environmental suitability into five categories: very high (81–99%, red), high (61–80%, orange), moderate (41–60%, yellow), low (21–40%, light green), and very low (≤20%, dark green).

To estimate the relative contribution of each variable, both percentage contribution (PC) and Jackknife analysis of AUC were applied [25]. The AUC index was used to evaluate model accuracy, where values > 0.9 indicate very high predictive performance, 0.8–0.9 indicate good performance, and 0.7–0.8 denote low model reliability. Presence data included historical records from northern Chile (Arica and Parinacota Region) and new records obtained in this study, with spatial verification performed to eliminate duplicates or spatial autocorrelation among points.

The resulting maps were interpreted based on their compatibility with Mediterranean-arid transitional agricultural regions, enabling the prediction of potential expansion areas and the implications of climate change for the regional distribution of this parasitoid wasp.

## 3. Results

### 3.1. Morphological Determination and Taxonomic Validation of Campsomeriella whitelyi in Chile’s Norte Chico

**Figure 1:**
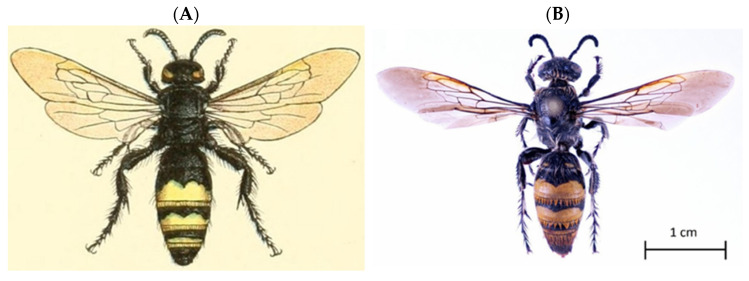
*Campsomeriella whitelyi* (Hymenoptera: Scoliidae). (**A**) Original illustration of the female, taken from the description by Kirby (1889) [17]; (**B**) recently collected female specimen from lowland areas of the Elqui Valley Basin, Coquimbo Region, Chile.

**Figure 2:**
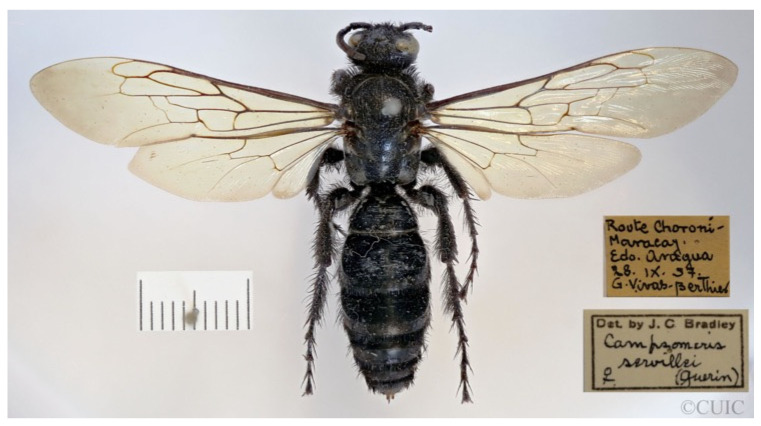
Dorsal view of female *Campsomeris servillei* (Guérin-Méneville, 1831) (Hymenoptera: Scoliidae). Image obtained from the Cornell University online database [26]. The labels shown correspond to the original specimen labels and are reproduced as in the original image.

The specific identification of the collected specimens was based on a detailed examination of external morphological characters recognized as diagnostic within the family Scoliidae, particularly in the tribe Campsomerini (Figure 1A). One of the most distinctive traits observed was the abdominal coloration pattern: the second and third tergites display a velvety black anterior region and a bright yellow posterior region, separated by a sharp transition line; the fourth tergite also bears a preapical yellow band (Figure 1A,B) [17,26,27]. This pattern is consistent with the original description of *Campsomeriella whitelyi* (Kirby, 1889) and allows for differentiation from morphologically similar species, such as *Campsomeris servillei* (Figure 2) [17,26], which lacks this coloration. Additionally, tergites 3–5 bear a basal band of yellow setae. The sixth segment is reddish, becoming more intense toward the apex, and shows a basal band of dark red setae. The ventral surface of the abdomen is black and glossy, with punctures arranged in transverse rows from which long, laterally oriented setae emerge—characters also consistent with previous diagnoses of the species (Figure 1B).

Among the complementary diagnostic characters used in the validation, the presence of a long, robust, and slightly curved posterior tibial spur—typical of the genus *Campsomeriella*—was noteworthy, as well as the characteristic wing venation: hyaline wings with a yellowish shade, ferruginous veins, and a rusty-brown border at the base. Body pilosity was abundant, with long gray setae on the head and thorax, reinforcing the species-level diagnosis. These observations were confirmed using regional morphological keys [6,10,18,19] and by direct comparison with reference specimens housed in Chile’s National Natural History Museum. The combination of these characters allowed for the confident validation of the specimens’ identity, ruling out the presence of other morphologically similar species within the Chilean and South American Scoliidae.

**Figure 3:**
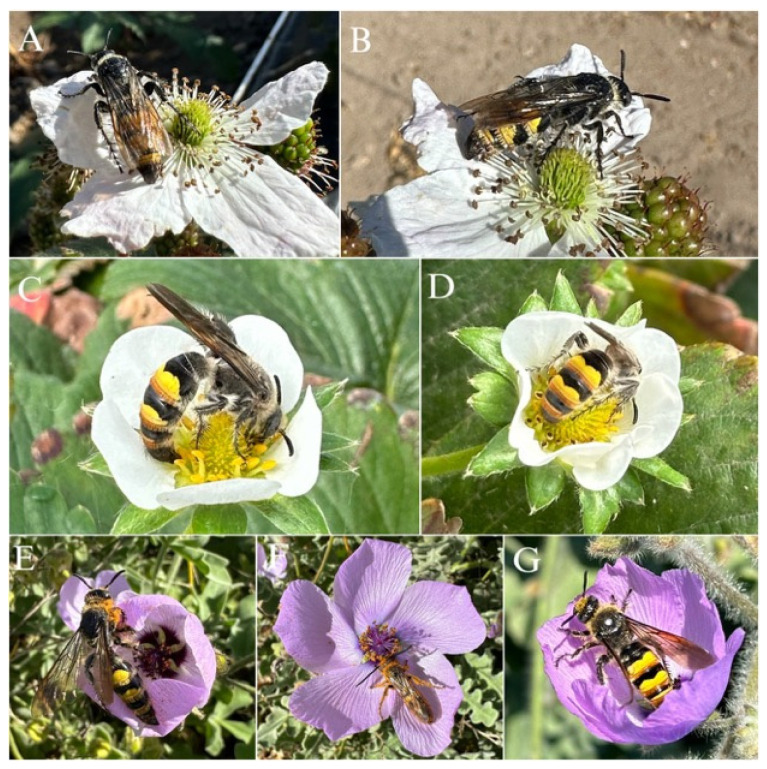
Adult specimens of *Campsomeriella whitelyi* (Hymenoptera: Scoliidae) collected in lowland areas of the Elqui Valley, Coquimbo Region, Chile. (**A**,**B**) Recorded on blackberry flowers (*Rubus ulmifolius* var. *fenomenal*); (**C**,**D**) strawberry flowers (*Fragaria × ananassa* var. *albion*); (**E**–**G**) malvilla flowers (*Cristaria glaucophylla*).

**Figure 4:**
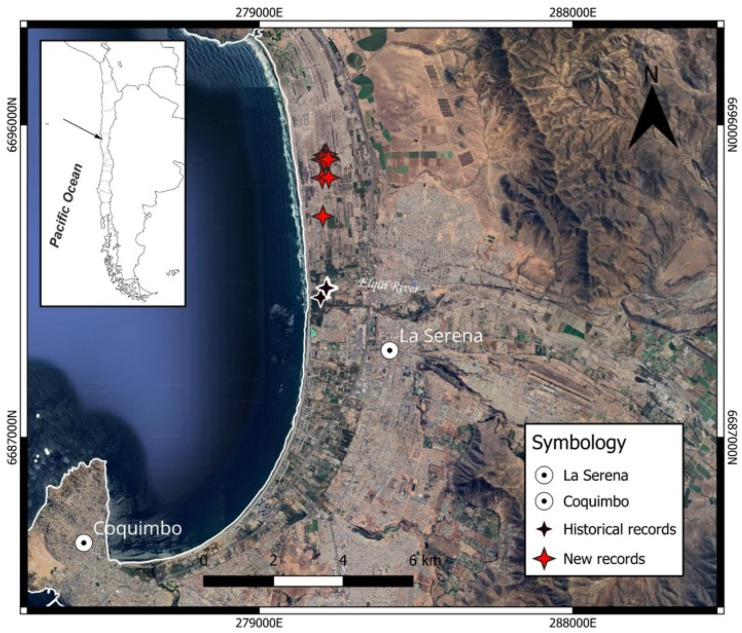
Distribution map of *Campsomeriella whitelyi* in Chile showing historical records (black) and new records in the Coquimbo Region (red).

Specimen collection was concentrated in areas cultivated with vegetables and minor fruits trees, including an agricultural land producing red fruits, among them *Rubus ulmifolius* (Schott, 1818), *Fragaria × ananassa* (Duchesne ex Weston, 1785), and the native species *Cristaria glaucophylla* Cavanilles, 1799 (Table 1, Figure 3 and Figure 4). The new distribution range of the species lies along the coastal strip of Elqui Province, between the mouth of the Elqui River and Punta de Teatinos, at an average altitude of 4 m a. s. l. Specimens were observed near dwellings and flying over exposed soil areas (Table 1), suggesting a close association with irrigated agricultural systems (Figure 3).

### 3.2. Evaluation of the Recent Distribution Pattern of Campsomeriella whitelyi in Terms of Ecological and Bioclimatic Factors

**Figure 5:**
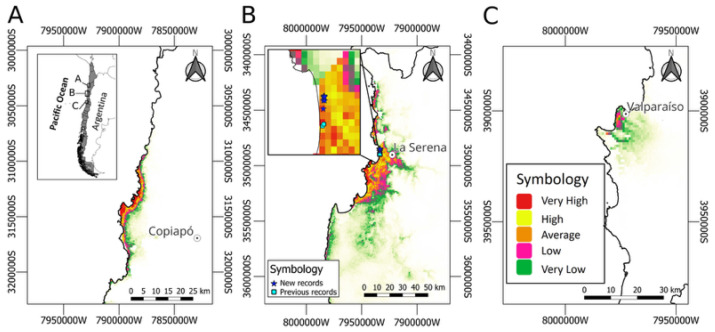
Potential distribution maps of *Campsomeriella whitelyi* in Chile’s Norte Chico, generated through MaxEnt modeling. (**A**) Predicted distribution from the Atacama region. (**B**) Predicted distribution from the Coquimbo region. (**C**) Predicted distribution for the Valparaíso region. Red indicates very high probability of occurrence; yellow, high probability; orange, moderate probability; pink, low probability; and green, very low probability. Yellow stars represent the records used in this study, including new localities in the Coquimbo Region.

The MaxEnt model results from 50 bootstrap replicates yielded an AUC = 0.998 ± 0.003, indicating excellent predictive performance but with some influence of the small sample size. High environmental suitability for the species was predicted at lower elevations in the coastal sector of the Atacama coast, whereas the Coquimbo Region exhibited the highest suitability values, represented on the map by orange to red tones. Overall, approximately 484 km^2^ was classified as areas of high or very high probability of occurrence, corresponding to suitability values between 0.6 and 1.0. This parameter was highest in the lowlands of the Elqui and Limarí communes and started declining about 1 km north of Huentelauquén. In the Valparaíso Region, low suitability values were recorded around Laguna Verde (Figure 5).

**Figure 6:**
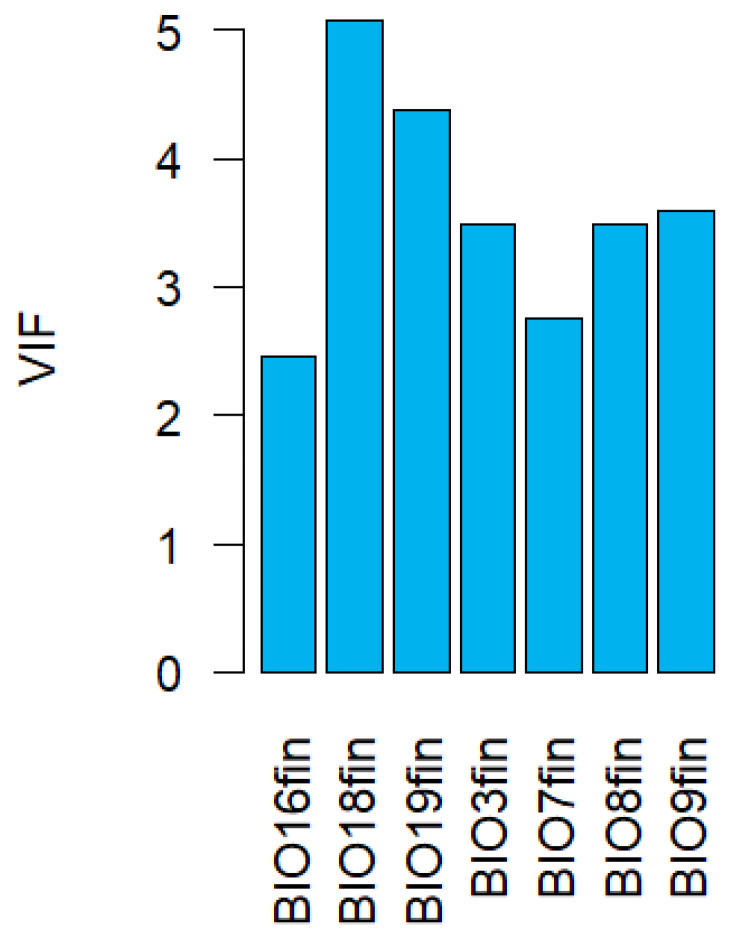
Variance inflation factor (VIF) values for the environmental variables considered in the MaxEnt model. Variables with VIF values above the selected threshold (VIF > 5) were considered highly collinear and excluded from the final model calibration.

**Figure 7:**
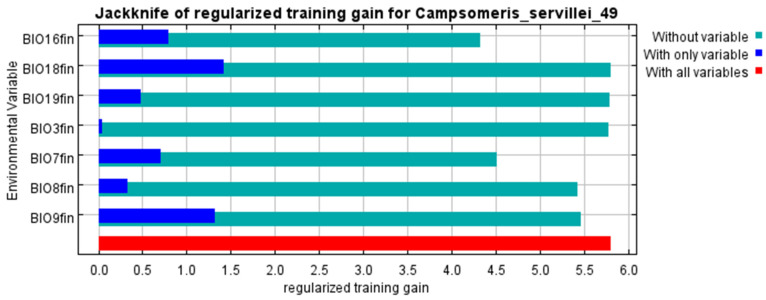
Jackknife test of variable importance based on training gain.

The environmental variables included in the final MaxEnt model were selected after assessing multicollinearity using the variance inflation factor (VIF), retaining only variables with VIF values below the selected threshold (Figure 6). The Jackknife test of training gain identified BIO18 as the variable contributing most to model performance when used in isolation, followed by BIO9, BIO16, and BIO7 (Figure 7). In contrast, the percent contribution analysis indicated that BIO7 had the highest explanatory power (41.2%), followed by BIO16 (35.4%) and BIO9 (17.6%) (Table 3). Permutation importance further highlighted BIO9 as a key variable (63.2%), despite its lower percent contribution, suggesting that it contains important independent information that is not shared with other predictors.

Overall, BIO7 (temperature annual range) and BIO16 (precipitation of wettest quarter) emerged as the main drivers of the model based on their high percent contributions. BIO9 (mean temperature of driest quarter) also played a significant role, particularly as indicated by its high permutation importance, suggesting that it acts primarily as a limiting environmental factor rather than as a direct driver of model gain. Although BIO18 (precipitation of warmest quarter) was highlighted by the Jackknife test when used in isolation, its low percent contribution and permutation importance indicate a minor overall influence on the model. Consequently, BIO18 was not considered a primary predictor of the species’ distribution.

## 4. Discussion

### 4.1. Taxonomic Validation of Collected Specimens

The morphological analysis of the scoliid wasps collected in coastal agroecosystems of the Coquimbo Region confirmed their identification as *Campsomeriella whitelyi*, a species previously reported only from the far north of Chile and originally described in the Tambo Valley, Peru [17]. Recent taxonomic revisions correcting earlier misassignments to the genus *Campsomeris* have improved the current understanding of Campsomerini diversity and distribution in Chile [10,11].

From a biogeographical perspective, this record constitutes a substantial southward extension of the known range of the species, expanding its distribution from the northern tip of Chile (Arica and Parinacota) to the north-central zone (Coquimbo Region). This finding supports the hypothesis that intermediate agricultural areas—particularly those with limited sampling coverage—may harbor underestimated entomological diversity [5]. Moreover, it highlights the need to include productive landscapes (agroecosystems) in systematic sampling efforts and biodiversity assessment programs, particularly in ecological and climatic transition areas, particularly in ecological and climatic transition areas.

### 4.2. Analysis of Recent Distribution Patterns Based on Ecological and Bioclimatic Variables

The occurrence of *Campsomeriella whitelyi* in semi-arid agroecosystems of Chile’s Norte Chico represents a notable case of spatial expansion under conditions contrasting with its historical distribution. These systems—characterized by seasonal water availability, moderate temperatures, and intensive agricultural landscapes—demonstrate the species’ remarkable ecological plasticity. Local persistence may be favored by the availability of potential scarabaeid hosts commonly associated with horticultural soils, such as Melolonthinae larvae, together with diverse floral resources and microclimatic stability associated with irrigation management [8]. Furthermore, the interregional transport of horticultural products, compost, and agricultural soils may contribute to passive dispersal processes in entomological fauna [15]. Although this mechanism was not directly assessed in the present study, it represents a plausible pathway of anthropogenic-assisted dispersal with potential implications for biodiversity conservation and plant health monitoring.

The use of ecological modeling tools, such as MaxEnt, provides a reliable approach to anticipate potential expansion zones and identify areas of high environmental suitability. Such approaches can guide surveillance strategies, delineate priority monitoring areas, and inform management planning under scenarios of climate change and land-use transformation [25].

### 4.3. Functional and Applied Implications

Beyond its systematic and biogeographical significance, *Campsomeriella whitelyi* stands out for its functional potential as a natural enemy of rhizophagous larvae. This attribute is particularly relevant in crops for which Scarabaeidae represent a major threat, such as potatoes, beets, and minor fruit trees [28,29].

The incorporation of native parasitoid species into integrated pest management (IPM) programs may constitute an effective and lower-risk alternative to the introduction of exotic Hymenoptera [7]. Nevertheless, the potential application of *C. whitelyi* in biological control strategies requires further studies addressing host associations, parasitism rates, reproductive ecology, and population dynamics.

This case highlights the importance of integrating taxonomic, biogeographic, and ecological approaches for improving the understanding and management of entomological biodiversity in semi-arid agroecosystems of Chile.

## 5. Conclusions

This study confirms the presence of *Campsomeriella whitelyi* in the Coquimbo Region, significantly extending its known distribution southward in Chile. Through taxonomic validation and georeferenced analysis, it was demonstrated that this species can establish in semi-arid coastal agroecosystems—from the Arica and Parinacota Region to the Valparaiso Region—likely facilitated by favorable climatic conditions and interregional agricultural transport.

The taxonomic confirmation of the collected specimens corrects previous misidentifications and integrates *Campsomeriella whitelyi* as a relevant component of the scoliid entomofauna of north-central Chile. This finding also reveals historical knowledge gaps in agricultural zones undergoing ecological transition, emphasizing the need to update taxa distributions through rigorous morphological revisions and targeted sampling efforts.

From an agroecological perspective, *Campsomeriella whitelyi* emerges as a promising candidate for integrated pest management (IPM) programs, particularly because of its efficacy as a parasitoid of Scarabaeidae larvae and its potentially low ecological impact. Its incorporation into biological control strategies could reduce pesticide dependence and promote sustainable agricultural practices. However, additional studies on its reproductive biology, parasitism rates, trophic interactions, and population dynamics are necessary to ensure its safe and effective application in Chilean agroecosystems.

Finally, this work emphasizes the importance of strengthening entomological monitoring networks, linking biological collections with applied research, and promoting functional conservation policies that acknowledge the role of biodiversity in maintaining productive-system stability. The use of ecological distribution models can further assist in anticipating potential areas of establishment and defining surveillance priorities under climate change scenarios.

## Figures and Tables

**Table 1:** Collection and sightings records of *Campsomeriella whitelyi* (Scoliidae) from the lower elevations of the Elqui Valley, Coquimbo Region, Chile.

ID	X	Y	Masl	Collected in/Associated to	Date	Collector
1	−29.891883, −71.270306	−29.891883, −71.270306	2	Peri-urban area	5 June 2017	Macarena González
2	−29.889300, −71.268331	−29.889300, −71.268331	3	Peri-urban area	5 June 2017	Macarena González
3	−29.889428, −71.268239	−29.889428, −71.268239	2	*Tessaria absinthioides*	4 June 2017	Macarena González
4	−29.889167, −71.268333	−29.889167, −71.268333	3	Sandy substrate (flying)	2 June 2017	Jaime Pizarro-Araya & F.M. Alfaro
5	−29.889722, −71.268333	−29.889722, −71.268333	2	Sandy substrate (flying)	2 June 2017	Jaime Pizarro-Araya & F.M. Alfaro
6	−29.889444, −71.268056	−29.889444, −71.268056	3	Sandy substrate (flying)	2 June 2017	Jaime Pizarro-Araya & F.M. Alfaro
7	−29.889444, −71.268333	−29.889444, −71.268333	3	Peri-urban area	15 April 2020	Jaime Pizarro-Araya
8	−29.889722, −71.268333	−29.889722, −71.268333	2	Peri-urban area	15 April 2020	Jaime Pizarro-Araya
9	−29.889444, −71.268611	−29.889444, −71.268611	3	Peri-urban area	15 April 2020	Jaime Pizarro-Araya
10	−29.855556, −71.267500	−29.855556, −71.267500	8	*Cristaria glaucophylla*	3 May 2024	Jaime Pizarro-Araya
11	−29.854722, −71.267778	−29.854722, −71.267778	7	*Cristaria glaucophylla*	18 May 2024	Macarena González
12	−29.884739, −71.268644	−29.884739, −71.268644	6	Sandy substrate (flying)	10 May 2024	Jaime Pizarro-Araya & J.E. Calderón
13	−29.856389, −71.268333	−29.856389, −71.268333	5	Sandy substrate (flying)	3 April 2025	Macarena González
14	−29.856389, −71.268333	−29.856389, −71.268333	5	Flying over the ground	4 April 2025	Macarena González
15	−29.885956, −71.267564	−29.885956, −71.267564	3	Flying over the ground	15 April 2025	Macarena González
16	−29.860833, −71.267222	−29.860833, −71.267222	8	Flying over the ground	24 April 2025	Macarena González

**Table 2:** Bioclimatic variables used in the distribution model of *Campsomeriella whitelyi* (Hymenoptera: Scoliidae). The variables correspond to climatic layers from the WorldClim v2.1 (30 arc-seconds) that also include annual and seasonal temperature and precipitation parameters used to estimate environmental suitability in Chile’s Norte Chico.

Name	Description
B1	Annual mean temperature
B2	Mean diurnal range [monthly mean (maximum temperature–minimum temperature)]
B3	Isotherm
B4	Temperature seasonality (standard deviation × 100)
B5	Maximum temperature of the warmest month
B6	Minimum temperature of the coldest month
B7	Annual temperature range (B5–B6)
B8	Mean temperature of the wettest quarter
B9	Mean temperature of the driest quarter
B10	Mean temperature of the warmest quarter
B11	Mean temperature of the coldest quarter
B12	Annual precipitation
B13	Precipitation of the wettest month
B14	Precipitation of the driest month
B15	Precipitation seasonality (coefficient of variation)
B16	Precipitation of the wettest quarter
B17	Precipitation of the driest quarter
B18	Precipitation of the warmest quarter
B19	Precipitation of the coldest quarter

**Table 3:** Contribution of bioclimatic variables to the distribution model of *Campsomeriella whitelyi* (Hymenoptera: Scoliidae). The table indicates the percentage contribution and permutation importance of each variable used in the MaxEnt model, based on records from northern Chile and the Coquimbo Region.

Variable	Percent Contribution	Permutation Importance
BIO18fin	41.6	0.1
BIO7fin	22.9	11.4
BIO19fin	13.9	10.3
BIO16fin	13.3	23
BIO3fin	4.3	1.8
BIO9fin	2.8	49.1
BIO8fin	1.2	4.2

## Data Availability

The original contributions presented in this study are included in the article. Further inquiries can be directed to the corresponding author.
